# Direction of Spontaneous Processes in Non-Equilibrium Systems with Movable/Permeable Internal Walls

**DOI:** 10.3390/e26080713

**Published:** 2024-08-22

**Authors:** Robert Hołyst, Paweł J. Żuk, Anna Maciołek, Karol Makuch, Konrad Giżyński

**Affiliations:** 1 Institute of Physical Chemistry, Polish Academy of Sciences, Kasprzaka 44/52, 01-224 Warszawa, Poland; kmakuch@ichf.edu.pl (K.M.); kgizynski@ichf.edu.pl (K.G.); 2 Max-Planck-Institut für Intelligente Systeme Stuttgart, Heisenbergstr. 3, D-70569 Stuttgart, Germany

**Keywords:** thermodynamics, non-equilibrium thermodynamics, gravity, stationary state, steady state, entropy

## Abstract

We consider three different systems in a heat flow: an ideal gas, a van der Waals gas, and a binary mixture of ideal gases. We divide each system internally into two subsystems by a movable wall. We show that the direction of the motion of the wall, after release, under constant boundary conditions, is determined by the same inequality as in equilibrium thermodynamics dU−đQ≤0. The only difference between the equilibrium and non-equilibrium laws is the dependence of the net heat change, đQ, on the state parameters of the system. We show that the same inequality is valid when introducing the gravitational field in the case of both the ideal gas and the van der Waals gas in the heat flow. It remains true when we consider a thick wall permeable to gas particles and derive Archimedes’ principle in the heat flow. Finally, we consider the Couette (shear) flow of the ideal gas. In this system, the direction of the motion of the internal wall follows from the inequality $dE-đQ-đW_{s}\leq 0$, where dE is the infinitesimal change in total energy (internal plus kinetic) and $đW_{s}$ is the infinitesimal work exchanged with the environment due to the shear force imposed on the flowing gas. Ultimately, we synthesize all these cases within a general framework of the second law of non-equilibrium thermodynamics.

## 1. Introduction

The direction of spontaneous processes follows the second law of equilibrium thermodynamics [1]. It states that isolated systems reaching equilibrium increase their entropy. The second law is practical. It allows us to predict which chemical reactions occur spontaneously, under what thermodynamic conditions liquids evaporate or freeze, and how to design efficient engines. However, such a law, although needed, has yet to be discovered for out-of-equilibrium systems characterized by the continuous flux of energy flowing across them. This remains true despite large efforts [2,3,4,5,6,7,8,9,10,11,12,13,14,15,16,17,18,19,20,21,22,23,24,25,26,27,28], and sucesses are limited to either isothermal situations or small temperature differences [21,22,23,24,25,26,27,28]. Promising results have been obtained in small-scale systems allowing for a discrete description [29,30], and they pave the way for a trajectory-based approach to macroscopic stochastic thermodynamics [31].

Non-equilibrium thermodynamics today (called the thermodynamics of irreversible processes) is a set of non-linear differential equations. Depending on the specific system under consideration, these equations include the conservation of mass, momentum, and energy [32,33,34], as well as charge conservation with Maxwell’s equations in the case of magnetohydrodynamics or the Poisson–Nernst–Planck equations for electrokinetics, among others. The conservation laws are typically accompanied by closing relations like the ideal gas equation of state. This paper will present preliminary observations for a few systems without macroscopic motion and for a single system with macroscopic motion. These results may provide insights toward formulating a general second law of non-equilibrium thermodynamics.

Non-equilibrium states involve macroscopic energy fluxes flowing across the system [33]. These fluxes are sustained within the system by non-vanishing gradients of temperature (in the case of heat flow), pressure (in the case of mass flow), or chemical potential (in the case of particle diffusion). Thus, a non-equilibrium state has non-uniform temperature, pressure, chemical potential, and velocity. The local equations of state, relating internal energy and pressure to density and temperature, and the spatial profiles of density, temperature, and concentrations fully characterize non-equilibrium states. In our recent papers, we formulated the first law of global thermodynamics for various non-equilibrium systems [35,36,37], including cases involving gravity [36] or Couette flow [38]. We represented internal energy as a function of a few global state parameters. These parameters were obtained by mapping a non-equilibrium, and by definition, non-uniform system into a uniform one. We averaged local equations of state over the system’s volume. This averaging resulted in global equations of state, which we wrote in the same form as at equilibrium. These global non-equilibrium equations of state included new state parameters. For example, the internal energy of the van der Waals gas subjected to continuous heat flux $U(S^{*},V,N,a^{*},b^{*})$ is a function of five state parameters, where $S^{*}$ is the non-equilibrium entropy, *V* is the volume, *N* is the number of particles, and $a^{*}$ and $b^{*}$ are new state parameters—renormalized van der Waals interaction parameters. The net heat that flows in/out of the system to change the internal energy of the van der Waals gas is given by:

(1) $$đQ=T^{*}dS^{*}-\frac{N^{2}}{V}da^{*}+Nk_{B}T^{*}{\left(\frac{V}{N}-b^{*}\right)}^{-1}db^{*}.$$

The $a^{*}$ and $b^{*}$ state parameters appear in the net heat differential because, in a non-uniform system, the change in the density profile leads to the local absorption or release of heat. In general, under the mapping construction of global thermodynamics, all material parameters in the equilibrium equations of states become state parameters of the non-equilibrium state.

In a system kept at a constant temperature at equilibrium, energy is exchanged with the environment as heat only. The second law of equilibrium thermodynamics states [1,39] that the Helmholtz free energy, F(T,V,N,x), is minimized with respect to *x* (the variable describing the internal constraint) at constant T,V, and *N* (temperature, volume, and number of particles, respectively). The minimization defines the equilibrium value of *x*. The change in *F* as a function of *x* is negative if the initial *x* does not correspond to the equilibrium state. Thus, the change in *F* when we move the system from the initial to the final *x* is given by ΔF≤0. Rewriting this equation using internal energy and entropy yields ΔU−TΔS≤0. In the infinitesimal form, we obtain dU−TdS≤0. Finally, the net heat (the heat that enters or leaves the system and changes the internal energy) is đQ=TdS. In general, the second law states that in spontaneous processes,

(2) dU−đQ≤0.

Thus, the system minimizes part of its internal energy. This part does not account for the energy, which, at equilibrium, is continuously exchanged with the environment (here, the net heat).

In this paper, we elucidate non-equilibrium systems in a continuous heat flow. Out of equilibrium, net heat becomes the amount of energy that enters or leaves the system in the form of heat and changes the internal energy. We show the consequences of applying dU−đQ≤0 in the systems’ stationary (steady) states. Section 2 discusses the ideal gas, van der Walls gas, and binary mixture of ideal gases enclosed by two fixed walls at different temperatures. We cite the functional forms of dU and đQ as functions of the state parameters for a single compartment [35,36,37] and point out that đQ differs from what we know from equilibrium. Next, as is done at equilibrium, we divide the enclosed gas with an internal diathermic wall and verify that (2) determines the spontaneous motion of the wall toward the stationary position. Section 3 extends the problem to account for gravity and heat flow. We present the energy differential and the fundamental relation for a single compartment in the case of the ideal gas [36] and expand them to the case of the van der Waals gas. Then, we introduce the internal diathermic wall between two compartments and verify that (2) holds during the spontaneous motion of the wall. Finally, we assume that the wall has a finite thickness and is permeable (porous). This leads to the derivation of Archimedes’ principle from the second law of thermodynamics. Section 4 demonstrates that the relative stability of multiple stable states can be compared within the proposed framework. We cite a system where the ideal gas is heated volumetrically [16] and calculate the integral of external work necessary to switch between the states. In Section 5, using the example of the ideal gas in shear flow [38], we introduce a further generalization of the proposed second law to the case where, in addition to heat, the system also exchanges energy in the form of work. The work originates from the shear forces imposed on the gas flow. We conclude this paper by generalizing the second law in the discussion section.

## 2. The Ideal Gas, Gas Mixtures, and van der Waals Gas in Heat Flow

**Figure 1:**
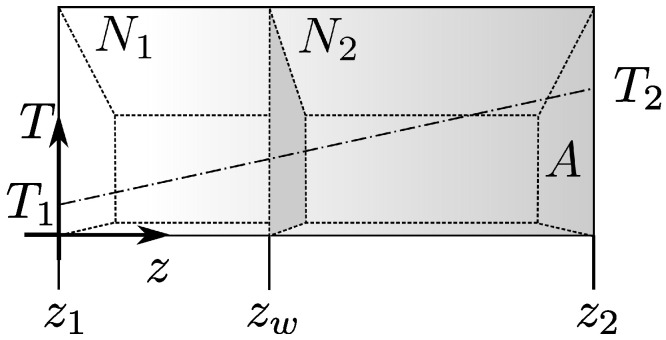
Schematic of a gas confined between two parallel walls with area A. The system is assumed to be translationally invariant in the *x* and *y* directions. The thin interior wall at $z_{w}$ is treated as an internal constraint. We assume that the internal wall is diathermic, impenetrable, and can move freely. The inner wall divides the system into two compartments containing $N_{1}$ and $N_{2}$ gas particles ($N_{1}+N_{2}=N$). The outer wall placed at $z_{1}=0$ has a fixed temperature $T_{1}$, and the outer wall placed at $z_{2}=L$ is kept at a fixed temperature $T_{2}>T_{1}$. The resulting linear temperature profile is indicated by the dash-dot line.

**Ideal gas.** The model geometry is defined by two parallel walls at $z_{1}=0$ and $z_{2}=L$, as shown in Figure 1. For now, let us focus on the arrangement that does not include the inner wall.

The area of each wall, *A*, is large enough so that the system is translationally invariant in the *x* and *y* directions. The walls are in contact with thermostats, which are maintained at different temperatures, $T_{1}<T_{2}$. In the presence of a heat flux, quantities specifying equilibrium thermodynamic states, such as number density n=N/V, pressure *p*, or temperature *T*, become space-dependent. The profiles n(z), p(z), and T(z) of these quantities can be determined using, for example, the irreversible thermo-hydrodynamics approach, which relies on the local equilibrium assumption and represents the conservation of mass, momentum, and energy, supplemented with the equations of states and relations between fluxes and thermodynamic forces. For a stationary state with a vanishing velocity field, the thermo-hydrodynamics problem reduces to the constant pressure condition $p\left(z\right)=p$.

The construction of global thermodynamics describing the non-equilibrium steady state is achieved by mapping it to a uniform system characterized by a finite number of state parameters. Mapping involves averaging the local pressure and energy over the system’s volume so that, when averaged, the global equations of state have the same form as in equilibrium. For an ideal gas with *f* degrees of freedom, this procedure gives, for a steady state with no mass flow,

(3) $$\begin{array}{cc} \; & p=\frac{1}{L}\int_{0}^{L}\;n\left(z\right)k_{B}T\left(z\right)dz=nk_{B}T^{*},\\ \; & U=\frac{f}{2}A\int_{0}^{L}\;n\left(z\right)k_{B}T\left(z\right)dz=\frac{f}{2}Vnk_{B}T^{*}, \end{array}$$

where $n=N/V=\left(1/L\right)\int_{0}^{L}n\left(z\right)dz$ is the average number density and $T^{*}$ is given by

(4) $$T^{*}=\frac{A\int_{0}^{L}dz\;n\left(z\right)T\left(z\right)}{A\int_{0}^{L}dz\;n\left(z\right)}.$$

The new state parameter $T^{*}$ can be interpreted as the average temperature of the uniform system onto which we have mapped the original, non-equilibrium one. The explicit expression for $T^{*}$ depends on the specific temperature and number density profile. In the case of constant pressure [35], the equations of irreversible thermo-hydrodynamics yield $T\left(z\right)=T_{1}+\left(T_{2}-T_{1}\right)\left(z/L\right)$, $n\left(z\right)=n\left(T_{2}-T_{1}\right)/\left[\log\left(T_{2}/T_{1}\right)T\left(z\right)\right]$, and as a result,

(5) $$T^{*}=\left(T_{2}-T_{1}\right)/\ln\left(T_{2}/T_{1}\right).$$

Because mapping gives us the same formal structure as we know from equilibrium, the variable $S^{*}$ conjugate to $T^{*}$ is given by

(6) $$S^{*}\left(U,V,N\right)/k_{B}=N\log\left[{\left(\frac{U}{\left(f/2\right)k_{B}N}\right)}^{f/2}\frac{V}{N}\right]+Ns_{0}/k_{B},$$

where the constant $s_{0}$ is chosen such that $S^{*}$ for $T_{2}=T_{1}$ gives the equilibrium expression for entropy [1]. The internal energy of a non-equilibrium steady state is thus a function of three state parameters $U\left(S^{*},V,N\right)$, with the thermodynamic relations

(7) $$\begin{array}{cc} {\left(\frac{\partial S^{*}}{\partial U}\right)}_{V,N} & =\frac{1}{T^{*}},\\ {\left(\frac{\partial S^{*}}{\partial V}\right)}_{U,N} & =\frac{p}{T^{*}}. \end{array}$$

**Binary mixture.** Mixing two ideal gases, *a* and *b*, with $f_{a}$ and $f_{b}$ degrees of freedom, respectively, adds to the temperature and density profile the concentration profile induced by a heat flux. As a result, the mapping procedure leads to a uniform system with two new state parameters. In addition to $T^{*}$, we have two effective degrees of freedom $f_{a}^{*}$ and $f_{b}^{*}$, which, however, are not independent. These parameters allow us to write the internal energy and pressure in the same form as in equilibrium:

(8) $$\begin{array}{cc} \; & p=n_{a}k_{B}T^{*}+n_{b}k_{B}T^{*},\\ \; & U=\frac{f_{a}^{*}}{2}Vn_{a}k_{B}T^{*}+\frac{f_{b}^{*}}{2}Vn_{b}k_{B}T^{*}, \end{array}$$

where $n_{a}=N_{a}/V$ and $n_{b}=N_{b}/V$ are the average number densities of the first and second components of the mixture, respectively. The effective degrees of freedom are obtained as

(9) $$\begin{array}{c} f_{i}^{*}=\frac{f_{i}}{x_{i}}\frac{1}{L}\int_{0}^{L}x_{i}\left(z\right)dz,\;i=a,b, \end{array}$$

where $x_{i}=n_{i}/n$ is the average number fraction of the *i* component and $x_{i}\left(z\right)$ is its profile; $x_{a}+x_{b}=1$ in the absence of chemical reactions. $f_{a}^{*}$ and $f_{b}^{*}$ are related via $\left(f_{a}^{*}/f_{a}\right)x_{a}+\left(f_{b}^{*}/f_{b}\right)x_{b}=1$. The non-equilibrium entropy $S^{*}$ has the same form as in equilibrium but with *T* replaced with $T^{*}$ and $f_{i}$ with $f_{i}^{*}$. It is the sum of the entropy of the two components of the mixture considered separately and the entropy of mixing:

(10) $$\begin{array}{cc} S^{*}\left(U,V,N_{a},N_{b},f_{a}^{*}\right)/k_{B} & =N_{a}\left[\frac{f_{a}^{*}}{2}+1+\ln\frac{V}{N_{a}}{\left(\frac{2U}{k_{B}\left(N_{a}f_{a}^{*}+N_{b}f_{b}^{*}\right)}\right)}^{f_{a}^{*}/2}\right]\\ \; & +N_{b}\left[\frac{f_{b}^{*}}{2}+1+\ln\frac{V}{N_{b}}{\left(\frac{2U}{k_{B}\left(N_{a}f_{a}^{*}+N_{b}f_{b}^{*}\right)}\right)}^{f_{b}^{*}/2}\right]\\ \; & -N_{a}\ln\frac{N_{a}}{N}-N_{b}\ln\frac{N_{b}}{N}+S_{0}/k_{B}. \end{array}$$

Here, $S_{0}$ may depend on $N_{a}$ and $N_{b}$. Again, we choose $S_{0}$ such that $S^{*}$ reduces to the equilibrium entropy of a binary mixture in the absence of heat flux. Thus, the internal energy of a binary mixture of ideal gases in the non-equilibrium steady state is a function of five state parameters: $U\left(S^{*},V,N_{a},N_{b},f_{a}^{*}\right)$.

**Van der Waals gas.** A gas of interacting particles obeying the van der Waals equations of state is described with two additional interaction parameters, *a* and *b*. As a result of the mapping procedure, we obtain

(11) $$\begin{array}{cc} \; & p=\frac{nk_{B}T^{*}}{1-nb^{*}}-a^{*}n^{2},\\ \; & U=\frac{f}{2}Vnk_{B}T^{*}-a^{*}Vn^{2}. \end{array}$$

Three new state parameters describe the van der Waals gas in the steady state. Besides the effective temperature $T^{*}$, defined by the same expression (4) as for the ideal gas, we have the effective interaction parameter $a^{*}$ given by

(12) $$a^{*}=\frac{1}{L}\int_{0}^{L}\frac{a\;n{\left(z\right)}^{2}}{n^{2}}dz,$$

and $b^{*}$ defined by the formula

(13) $$\frac{nk_{B}T^{*}}{1-nb^{*}}=\frac{1}{L}\int_{0}^{L}\frac{n\left(z\right)k_{B}T\left(z\right)}{1-bn(z)}dz.$$

Because Equation (11) has the same structure as in equilibrium, the non-equilibrium entropy $S^{*}$ has the same form as in equilibrium but with *T* replaced with $T^{*}$, *a* with $a^{*}$, and *b* with $b^{*}$. Specifically,

(14) $$S^{*}\left(U,V,N,a^{*},b^{*}\right)/k_{B}=N\log\left[{\left(\frac{U+\frac{a^{*}N^{2}}{V}}{\left(f/2\right)k_{B}N}\right)}^{f/2}\frac{V-Nb^{*}}{N}\right]+Ns_{0}/k_{B}.$$

Thus, the internal energy of the van der Waals gas in the non-equilibrium steady state is a function of five state parameters: $U\left(S^{*},V,N,a^{*},b^{*}\right)$.

Note that for all systems discussed above, the non-equilibrium entropy $S^{*}$ is part of the total entropy $S_{tot}$, defined as the integral of the volume entropy density s(z) over the system volume. It contains information about the heat absorbed/released in the system in addition to the dissipative background (temperature profile).

**Net heat.** In the case of a very slow transition between stationary states, e.g., by a slight change in temperature $T_{2}$ or a change in the distance between the confining walls *L*, the energy changes only through mechanical work and heat flow:

(15) dU=đQ+đW,withđW=−pdV,

where đQ is the net heat transferred to the system during a small change between two non-equilibrium stationary states. Thus, the above equation can be considered as the first law of non-equilibrium thermodynamics. Using the fundamental relations for each of the above examples (Equations (6), (10) and (14)), we can determine the net heat from the energy balance dU=đQ−pdV. For an ideal gas, we have

(16) $$dU=T^{*}dS^{*}-pdV,\;\mathrm{and}\;đQ=T^{*}dS^{*},$$

which has the same formal structure as in equilibrium.

This is different for a binary mixture of ideal gases, where the net heat acquires additional terms. In general,

(17) $$dU=T^{*}dS^{*}-pdV+\mu_{a}^{*}dN_{a}+\mu_{b}^{*}dN_{b}+F_{a}df_{a}^{*}+F_{b}df_{b}^{*},$$

and for a fixed number of components $N_{a}$ and $N_{b}$, we have

(18) $$đQ=T^{*}dS^{*}+F_{a}df_{a}^{*}+F_{b}df_{b}^{*},$$

where $df_{b}^{*}=-\frac{x_{b}f_{a}}{x_{a}f_{b}}df_{a}^{*}$ and $\frac{F_{i}}{T^{*}}=-{\left(\frac{\partial S^{*}}{\partial f_{i}^{*}}\right)}_{U,V,N_{a},N_{b},f_{j\neq i}^{*}}$, with $S^{*}$ given by Equation (10).

Additional terms in the net heat also occur for the van der Waals gas, where

(19) $$dU=T^{*}dS^{*}-pdV-\frac{N^{2}}{V}da^{*}+nk_{B}T^{*}{\left(\frac{V}{N}-b^{*}\right)}^{-1}db^{*},$$

so that

(20) $$đQ=T^{*}dS^{*}-\frac{N^{2}}{V}da^{*}+nk_{B}T^{*}{\left(\frac{V}{N}-b^{*}\right)}^{-1}db^{*}.$$

**Movable internal wall.** We introduce into the considered systems an internal constraint in the form of a wall parallel to the bounding walls (Figure 1). We assume the wall is thin, freely movable, diathermal, and impenetrable. The internal constraint divides the system into two subsystems, 1 and 2, each with a fixed number of particles, $N_{1}$ and $N_{2}$, and volumes, $V_{1}$ and $V_{2}$. We choose the volume of one subsystem, $V_{1}$, as the parameter representing the constraint. The total energy is the sum of the energies of the two subsystems:

(21) $$U\left(S_{1}^{*},V_{1},N_{1},S_{2}^{*},V-V_{1},N_{2},\ldots \right)=U_{1}\left(S_{1}^{*},V_{1},N_{1},\ldots \right)+U_{2}\left(S_{2}^{*},V-V_{1},N_{2},\ldots \right),$$

where … denotes possible other new state variables, such as $f_{a}^{*}$ for the binary mixture of ideal gases or $a^{*}$ and $b^{*}$ for the van der Waals gas. The steady position of the wall, which moves without friction, is determined by the equality of the pressures exerted by each subsystem: $p_{1}=p_{2}$.

In the case of a monoatomic ideal gas, the effective temperatures of both subsystems are [35]

(22) $$\begin{array}{c} T_{1}^{*}=\frac{\frac{V_{1}}{V}\left(T_{2}-T_{1}\right)}{\log\left(\frac{T_{1}+\frac{V_{1}}{V}\left(T_{2}-T_{1}\right)}{T_{1}}\right)},\;T_{2}^{*}=\frac{\left(1-\frac{V_{1}}{V}\right)\left(T_{2}-T_{1}\right)}{\log\left[\frac{T_{2}}{T_{1}+\frac{V_{1}}{V}\left(T_{2}-T_{1}\right)}\right]}. \end{array}$$

Using the explicit forms of $dU_{1},dU_{2}$ and $đQ_{1},đQ_{2}$, we can see that this condition can be obtained from the following minimum principle:

(23) $$dU_{1}+dU_{2}-đQ_{1}-đQ_{1}=-\left(p_{1}-p_{2}\right)dV_{1}\leq 0\;,$$

which we call the second law of non-equilibrium thermodynamics. Thus, the difference between the total energy of the system and the heat exchanged with the environment is minimized while the internal wall moves to the new position. The equality defines the condition of the stationary position of the wall, given by the equality of pressures $p_{1}=p_{2}$ in the two subsystems. For the specific case of an ideal gas, we have

(24) $$p_{1}=k_{B}\frac{N_{1}}{V}\frac{T_{2}-T_{1}}{\ln\left[\frac{T_{1}+\left(T_{2}-T_{1}\right)\frac{V_{1}}{V}}{T_{1}}\right]},\;\;p_{2}=k_{B}\frac{N_{2}}{V}\frac{T_{2}-T_{1}}{\ln\left[\frac{T_{2}}{T_{1}+\left(T_{2}-T_{1}\right)\frac{V_{1}}{V}}\right]}.$$

Demanding $p_{1}=p_{2}$ and solving for $V_{1}$ gives us the position of the internal wall at the stationary state for given temperatures $T_{2}$ and $T_{1}$:

(25) $$\frac{V_{1}}{V}=\frac{{\left(\frac{T_{2}}{T_{1}}\right)}^{N_{2}/N}-1}{\frac{T_{2}}{T_{1}}-1}.$$

We note that for the diathermal internal wall, there is only a single solution for $V_{1}$ for all possible values of $T_{2}>T_{1}$. In Section 4, we consider an adiabatic internal wall with volumetric heating and show that, in this case, there might be more solutions for $V_{1}$, among which we can compare stability.

## 3. Gravitational Field: Ideal Gas, van der Waals Gas, and Archimedes’ Principle

**Figure 2:**
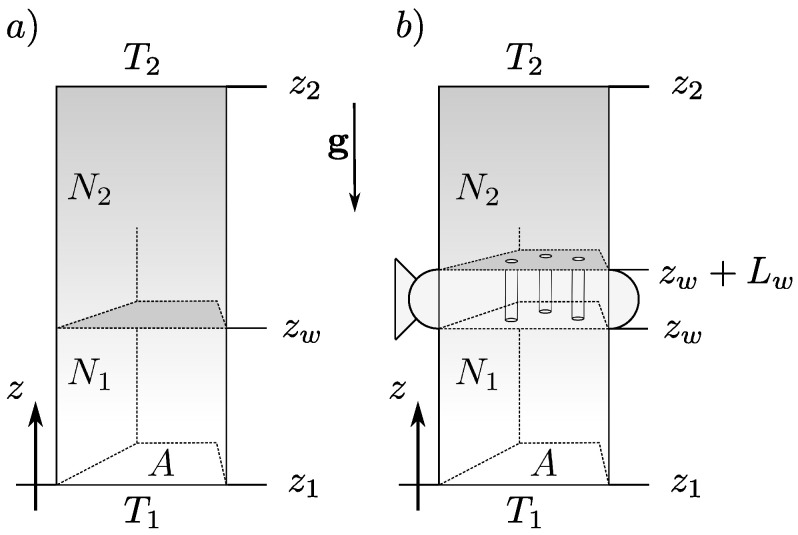
Gas column in gravity. A column of height *L* contains *N* moles of gas particles. The base ($z_{1}$) and top ($z_{2}$) are in contact with temperature reservoirs kept at two different temperatures: $T_{1}$ at the base, and $T_{2}$ at the top. The column’s base and top surface area equal *A*, and the system is translationally invariant in the *x* and *y* directions. (**a**) Impermeable and diathermal wall at $z_{w}$ separates the bottom and top sections with $N_{1}$ and $N_{2}$ particles of gas ($N_{1}+N_{2}=N$). (**b**) Permeable, thick ($L_{w}$) diathermal wall at $z_{w}$ separates sections containing $N_{1}$ and $N_{2}$ particles of gas ($N_{1}+N_{2}=N$).

**Ideal gas in gravity.** We insert the gas column into a constant, external gravitational field $-{\hat{e}}_{z}g$ that acts in the direction of the heat flux (see Figure 2). Here, the situation of interest is without macroscopic fluid motion, which means there is no Rayleigh–Benard convection [40,41,42]. The meanings of symbols from previous sections remain valid. The non-equilibrium entropy of a single gas column of an ideal gas is [36]

(26) $$S^{*}=\frac{3}{2}Nk_{B}\ln\frac{U-\frac{NM^{*}gL}{2}-NMgz_{1}}{U_{0}-\frac{NM_{0}gL_{0}}{2}-NMgz_{1,0}}+Nk_{B}\ln\frac{V}{V_{0}}+S_{0},$$

where $L=z_{2}-z_{1}$ is the height of the gas column; $z_{1}$ is the coordinate of the column base; $z_{2}$ is the coordinate of the column top; V=LA is the volume of the column, with *A* being the surface area of the column base; and *M* denotes the molecular mass of the gas. The lower index 0 indicates reference values. In the above, the state parameter $M^{*}$ is the renormalized mass defined with the help of the potential gravitational energy of the gas [36]

(27) $$E_{\mathrm{pot}}=\frac{NM^{*}gL}{2}\Leftrightarrow M^{*}=\frac{2E_{\mathrm{pot}}}{NgL},$$

which is contained inside the column

(28) $$E_{\mathrm{pot}}=gAL^{2}\int_{z_{1}/L}^{z_{2}/L}\rho z^{\prime }dz^{\prime }.$$

The state parameter $M^{*}$ is coupled to the gravitational field and informs about the system’s potential energy with respect to the position of the column base. To account for all effects of gravity, we also need to include the potential energy of the base of the column

(29) $$E_{\mathrm{pot},0}=NMgz_{1}.$$

The total internal energy of the gas column

(30a) $$U=\frac{3}{2}Nk_{B}T^{*}+\frac{NM^{*}gL}{2}+NMgz_{1},$$

consists of thermal and gravitational contributions. The thermal contribution (through the mapping procedure) defines the renormalized temperature $T^{*}$

(30b) $$E_{T}=\frac{3}{2}Nk_{B}T^{*}=AL\int_{z_{1}/L}^{z_{2}/L}\frac{3k_{B}}{2M}\rho Tdz^{\prime }\Leftrightarrow T^{*}=\frac{V}{NM}\int_{z_{1}/L}^{z_{2}/L}\rho Tdz^{\prime }.$$

The average pressure inside the column is the same as in the case without gravity (3)

(30c) $$p^{*}=\frac{Nk_{B}T^{*}}{V}.$$

This is because changing the surface area of the column base does not affect the gravitational energy, and all work is done against the thermal motion of gas particles. However, the presence of an external field introduces anisotropy in the pressure profile. The pressures at the top

(30d) $$p\left(z_{2}\right)=\frac{Nk_{B}T^{*}}{V}-\frac{NM^{*}gL}{2V},$$

and bottom

(30e) $$p\left(z_{1}\right)=p\left(z_{2}\right)+\frac{NMgL}{V}$$

of the column are different. The presented formulas are consistent with the functional form of $S^{*}$ (26) and the internal energy differential

(31) $$\begin{array}{cc} dU= & T^{*}dS^{*}+N\frac{gL}{2}dM^{*}-\left(\frac{Nk_{B}T^{*}}{V}-\frac{NM^{*}gL}{2V}\right)\frac{V}{L}dL-\frac{Nk_{B}T^{*}}{V}dV+NMgdz_{1}\\ = & T^{*}dS^{*}+N\frac{gL}{2}dM^{*}+đW=đQ+đW. \end{array}$$

In the above, we distinguish two separate ways of changing the internal energy. The first is through heat,

(32a) $$đQ=T^{*}dS^{*}+N\frac{gL}{2}dM^{*},$$

which includes entropic components and gravitational interactions between the external field and the mass inside the column. The second is through mechanical interactions exerted on the system boundaries through mechanical work

(32b) $$đW=-\left(\frac{Nk_{B}T^{*}}{V}-\frac{NM^{*}gL}{2V}\right)\frac{V}{L}dL-\frac{Nk_{B}T^{*}}{V}dV+NMgdz_{1}.$$

We deliberately wrote the work differential to distinguish between the column elongation, changes in the column volume, and changes in the column base position. The column elongation is coupled to the pressure at its top, while volume changes affect the average pressure.

In the two-compartment system (Figure 2a), the bottom part contains $N_{1}$ particles, and the top part contains $N_{2}$ particles of an ideal gas. We separate the compartments with a thin, diathermal wall at $z=z_{w}$. We assume there are no changes in the column cross-sectional area (dA=0) and set this contribution to the energy differential to 0. We rewrite the energy balances for the bottom ($U_{1}$) and top ($U_{2}$) segments explicitly to account for the displacement of the separating wall

(33a) $$\begin{array}{cc} dU_{1}= & T_{1}^{*}dS_{1}^{*}+N_{1}\frac{g(z_{w}-z_{1})}{2}dM_{1}^{*}-\left(\frac{N_{1}k_{B}T_{1}^{*}}{V_{1}}-\frac{N_{1}M_{1}^{*}g\left(z_{w}-z_{1}\right)}{2V_{1}}\right)\frac{V_{1}}{\left(z_{w}-z_{1}\right)}dz_{w}\\ = & T_{1}^{*}dS_{1}^{*}+N_{1}\frac{g(z_{w}-z_{1})}{2}dM_{1}^{*}-p_{1}\left(z_{w}\right)A\;dz_{w}=đQ_{1}+đW_{1}, \end{array}$$

(33b) $$\begin{array}{cc} dU_{2}= & T_{2}^{*}dS_{2}^{*}-N_{2}\frac{g(z_{2}-z_{w})}{2}dM_{2}^{*}+\left(\frac{N_{2}k_{B}T_{2}^{*}}{V_{2}}-\frac{N_{2}M_{2}^{*}g\left(z_{2}-z_{w}\right)}{2v_{2}}\right)\frac{V_{2}}{\left(z_{2}-z_{w}\right)}dz_{w}+N_{2}Mgdz_{w}\\ = & T_{2}^{*}dS_{2}^{*}+N_{2}\frac{g(z_{2}-z_{w})}{2}dM_{2}^{*}+p_{2}\left(z_{2}\right)A\;dz_{w}+N_{2}Mgdz_{w}=đQ_{2}+đW_{2}. \end{array}$$

The sign in front of $p_{2}$ is consistent with the direction of changes in $dz_{w}$. Following the second law of thermodynamics, we check whether the change in internal energies, when subtracted from all energies exchanged with the environment during the process, leads to the proper spontaneous state of the system, even beyond equilibrium. For the column subject to gravity with a fixed base and top, this is represented by the heat balance

(34) $$\begin{array}{c} dU_{1}+dU_{2}-đQ_{1}-đQ_{2}=\left(A\;\left(p_{2}\left(z_{2}\right)-p_{1}\left(z_{w}\right)\right)+N_{2}Mg\right)dz_{w}\leq 0. \end{array}$$

We find that the wall will position itself where the force necessary to expand the bottom part of the column balances the sum of the forces required to compress the top part of the column and, additionally, to raise its weight.

**Van der Waals gas in gravity.** We combine the results from the previous sections and construct an analogous description of the van der Waals gas in a gravitational field. For the single compartment, the stationary entropy is

(35) $$S^{*}=\frac{3}{2}Nk_{B}\ln\frac{U-\frac{NM^{*}gL}{2}-NMgz_{1}+a^{*}\frac{N^{2}}{V}}{U_{0}-\frac{N_{0}M_{0}gL_{0}}{2}-N_{0}Mgz_{1,0}+a_{0}^{*}\frac{N_{0}^{2}}{V_{0}}}+Nk_{B}\ln\frac{V-Nb^{*}}{V_{0}-N_{0}b_{0}^{*}}+S_{0}.$$

The internal energy of the van der Waals gas in a gravitational field is given by

(36a) $$U=\frac{3}{2}Nk_{B}T^{*}+\frac{NM^{*}gL}{2}+NMgz_{1}-a^{*}\frac{N^{2}}{V}.$$

The pressures are as follows:

(36b) $$p_{\mathrm{av}}=\frac{Nk_{B}T^{*}}{V-Nb^{*}}-a^{*}\frac{N^{2}}{V^{2}}$$

is the average pressure,

(36c) $$p\left(z_{2}\right)=\frac{Nk_{B}T^{*}}{V-Nb^{*}}-a^{*}\frac{N^{2}}{V^{2}}-\frac{NM^{*}gL}{2V}$$

is the pressure at the top of the column, and

(36d) $$p\left(z_{1}\right)=p\left(z_{2}\right)+\frac{NMgL}{V}$$

is the pressure at the bottom of the column. The energy differential has the form

(37) $$\begin{array}{cc} dU= & T^{*}dS^{*}+N\frac{gL}{2}dM^{*}-\left(\frac{Nk_{B}T^{*}}{V-Nb^{*}}-a^{*}\frac{N^{2}}{V^{2}}-\frac{NM^{*}gL}{2V}\right)\frac{V}{L}dL\\ \; & -\left(\frac{Nk_{B}T^{*}}{A\left(V-Nb^{*}\right)}-a^{*}\frac{N^{2}}{V^{2}}\right)dV-\frac{N^{2}}{V}da^{*}+Nk_{B}T^{*}{\left(\frac{V}{N}-b^{*}\right)}^{-1}db^{*}\\ \; & +NMgdz_{1}\\ = & T^{*}dS^{*}+N\frac{gL}{2}dM^{*}-\frac{N^{2}}{V}da^{*}+Nk_{B}T^{*}{\left(\frac{V}{N}-b^{*}\right)}^{-1}db^{*}+đW\\ \; & =đQ+đW, \end{array}$$

where we distinguish the heat differential, including the gravitational effects,

(38a) $$đQ=T^{*}dS^{*}+N\frac{gL}{2}dM^{*}-\frac{N^{2}}{V}da^{*}+Nk_{B}T^{*}{\left(\frac{V}{N}-b^{*}\right)}^{-1}db^{*},$$

and the mechanical work

(38b) $$đW=-\left(\frac{Nk_{B}T^{*}}{V-Nb^{*}}-a^{*}\frac{N^{2}}{V^{2}}-\frac{NM^{*}gL}{2V}\right)\frac{V}{L}dL-\left(\frac{Nk_{B}T^{*}}{A\left(V-Nb^{*}\right)}-a^{*}\frac{N^{2}}{V^{2}}\right)dV+NMgdz_{1}.$$

Likewise, for the ideal gas, we write the second law of stationary thermodynamics as

(39) $$\begin{array}{c} dU_{1}+dU_{2}-đQ_{1}-đQ_{2}=\left(A\;\left(p_{2}\left(z_{2}\right)-p_{1}\left(z_{w}\right)\right)+N_{2}Mg\right)dz_{w}\leq 0. \end{array}$$

Again, it reads that the wall will rest where the force necessary to extend the bottom compartment matches the force necessary to compress the top compartment plus the force to lift its weight.

**Archimedes’ principle.** Novel phenomena appear when the wall separating compartments is permeable. Assume that the wall has a finite thickness $L_{w}$ with its own mass $M_{b}$ and is pierced with small channels (pores) of negligible volume that allow for the passage of gas molecules (Figure 2b). We parametrize the position of the bottom of the wall at $z_{w}$. The porous wall separating the two compartments constitutes a separate thermodynamic system. We make additional assumptions regarding the wall: the gas that fills the pores inside the wall has a volume $V_{3}\ll V_{2},V_{1}$, but each pore is large enough so that the gas is in the thermal (not Knudsen) regime, and the wall is an excellent thermal conductor (temperature is constant inside the wall).

Each compartment containing a perfect gas is described by the fundamental relation (35). Since we allow for particle exchange, the energy balance (37) has to account for the resulting changes in energy $\frac{\partial U}{\partial N}dN=\mu^{*}dN$. We do not elucidate this term and treat it formally because it will cancel out during further derivation. The amended energy differential is

(40) $$\begin{array}{cc} dU= & T^{*}dS^{*}+\frac{NgL}{2}dM^{*}-\left(\frac{Nk_{B}T^{*}}{V}-\frac{NM^{*}gL}{2V}\right)\frac{V}{L}dL+NMgdz_{1}+\mu^{*}dN\\ = & T^{*}dS^{*}+N\frac{gL}{2}dM^{*}+đW+\mu^{*}dN=đQ+đW+\mu^{*}dN. \end{array}$$

We rewrite the energy differential explicitly for both compartments. Compartment 1 is at the bottom, so its $z_{1}$ stays fixed while $z_{1}=z_{w}+L_{w}$ of the upper compartment 2 is subject to change:

(41a) $$\begin{array}{cc} dU_{1}= & T_{1}^{*}dS_{1}^{*}+N_{1}\frac{gL_{1}}{2}dM_{1}^{*}-\left(\frac{N_{1}k_{B}T_{1}^{*}}{V_{1}}-\frac{N_{1}M_{1}^{*}gL_{1}}{2V_{1}}\right)\frac{V_{1}}{z_{w}-z_{1}}dz_{w}+\mu_{1}^{*}dN, \end{array}$$

(41b) $$\begin{array}{cc} dU_{2}= & T_{2}^{*}dS_{2}^{*}+N_{2}\frac{gL_{2}}{2}dM_{2}^{*}+\left(\frac{N_{2}k_{B}T_{2}^{*}}{V_{2}}-\frac{N_{2}M_{2}^{*}gL_{2}}{2V_{2}}\right)\frac{V_{1}}{z_{2}-z_{w}-L_{w}}dz_{w}+N_{2}Mgdz_{w}-\mu_{2}^{*}dN. \end{array}$$

Here, we set $dL=dz_{w}$ and $dN=dN_{1}=-dN_{2}$, which results from the direction of motion of the platform and the flux of the gas.

This case is different from the previous situations because, apart from the motion of the wall, we also allow for the exchange of particles, which introduces an additional degree of freedom. As previously guided by the second law of equilibrium thermodynamics, we sought to subtract from the total energy contributions exchanged with the environment. We perform the following heuristic consideration to elucidate the structure of the emerging second law of steady-state thermodynamics.

To write the total energy balance during the process of wall motion, we need to calculate the energetic cost of a passage of a particle batch dN from compartment 1 to compartment 2 through the porous wall. One way to achieve this is to use the metaphor of an external agent, similar to Maxwell’s demon. We calculate the energy the demon has to spend to displace dN particles from the bottom to the top compartment. The passage starts when dN particles leave compartment 1, which generates an energy gain in the demon’s account:

(42) $$đE_{b1w}=-\mu_{1}^{*}dN.$$

Inserting dN particles into the pore end at temperature $T(z_{w})$ requires, in addition to the volumetric work, the thermal energy

(43) $$đE_{w1p}=-\frac{3}{2}k_{B}T\left(z_{w}\right)dN,$$

which the demon has to subtract from the account. Transition through the region border is accompanied by the volumetric work performed on both sides of the border. When a batch of dN leaves compartment 1, the remaining gas has to fill the space. Inside the pore, space for the incoming batch has to be accommodated. Both processes—expansion and compression—happen under the same temperature and pressure, which are continuous over the border. Therefore, having the same magnitude with opposite signs, they cancel out in the demon’s account. Next, the batch is pushed through the pore, and during that process, it changes the potential energy,

(44) $$đE_{w,\mathrm{pot}}=-MgL_{w}dN,$$

and undergoes decompression, performing work

(45) $$đW_{w}=-\left(\int_{z_{w}}^{z_{w}+L_{w}}p\left(z\right)\frac{\partial \left(\frac{V}{N}\left(z\right)\right)}{\partial z}dz\right)dN.$$

Both quantities need to be supplied by the demon and thus subtracted from the account. Once the end of the pore is reached, thermal energy

(46) $$đE_{w2p}=\frac{3}{2}k_{B}T\left(z_{w}+L_{w}\right)dN$$

is released and added to the demon’s account. Simultaneously, the particle needs to equilibrate to the bulk conditions by taking energy

(47) $$đE_{b2w}=\mu_{2}^{*}dN,$$

which has to be paid by the demon. During passage through the region border, again, space has to be emptied and filled on both sides of the pore end at $T(z_{w}+L_{w})$, which does not influence the demon’s account. The final state of the account and, thus, the energetic cost of the passage results from summing all the listed effects:

(48) $$\begin{array}{cc} \; & đE_{\mathrm{pass}}=\left(\mu_{2}^{*}-\mu_{1}^{*}+MgL_{w}\right)dN+\frac{3}{2}k_{B}\left(T\left(z_{w}+L_{w}\right)-T\left(z_{w}\right)\right)dN+W_{w}dN. \end{array}$$

To complete the energy balance, we also need to account for the potential energy change due to the motion of the mass of the wall $M_{w}$ in the gravitational field:

(49) $$đE_{w,\mathrm{pot}}=M_{w}gdz_{w}.$$

With the use of $đE_{\mathrm{pass}}$ and $đE_{w,\mathrm{pot}}$, we can write the second law of stationary thermodynamics:

(50) $$\begin{array}{cc} dU_{1}+dU_{2} & +đE_{w,\mathrm{pot}}-đQ_{1}-đQ_{2}+đE_{\mathrm{pass}}\\ \; & =\left(A\;\left(p_{2}\left(z_{w}+L_{w}\right)-p_{1}\left(z_{w}\right)\right)+M_{w}g\right)dz_{w}\\ \; & +\left(\frac{3}{2}k_{B}\left(T\left(z_{w}+L_{w}\right)-T\left(z_{w}\right)\right)-MgL_{w}+W_{w}\right)dN\leq 0. \end{array}$$

The first three terms represent the changes in the system. The following two terms represent the fluxes of energies that can be exchanged with the environment, and the last term accounts for the additional energetic costs to pass through the obstacle. We stress that all terms involving the chemical potential μ are canceled out.

To calculate $W_{w}$, we use the assumption that the wall is an excellent heat conductor so that $T\left(z_{w}+L_{w}\right)=T\left(z_{w}\right)$. This means that the demon had to support the cost of the isothermal expansion

(51) $$W_{w}=-k_{B}T\left(z_{w}\right)\ln\frac{p_{2}\left(z_{w}+L_{w}\right)}{p_{1}\left(z_{w}\right)},$$

which we can explicitly insert into the second law of stationary thermodynamics:

(52) $$\begin{array}{cc} \; & dU_{1}+dU_{2}+đE_{w,\mathrm{pot}}-đQ_{1}-đQ_{2}+đE_{\mathrm{pass}}\\ \; & =\left(A\;\left(p_{2}\left(z_{w}+L_{w}\right)-p_{1}\left(z_{w}\right)\right)+M_{w}g\right)dz_{w}-k_{B}T\left(z_{w}\right)\left(\ln\frac{p_{2}\left(z_{w}+L_{w}\right)}{p_{1}\left(z_{w}\right)}+\frac{MgL_{w}}{k_{B}T\left(z_{w}\right)}\right)dN\leq 0. \end{array}$$

From the first bracket, one can see that the wall will move until the pressure at the top of the bottom compartment (1) matches the pressure at the bottom of the top compartment (2) plus the weight of the wall divided by its surface area:

(53) $$p_{1}\left(z_{w}\right)-p_{2}\left(z_{w}+L_{w}\right)=\frac{M_{w}g}{A}.$$

This is the same as saying that the wall will move until the force necessary to expand the bottom compartment matches the force necessary to compress the top compartment plus the weight of the top compartment ($p_{2}\left(z_{w}+L_{w}\right)=p_{2}\left(z_{2}\right)+\frac{N_{2}Mg}{A}$; see (36d)) plus the weight of the wall. The second bracket informs about the conditions for the gas flow through the pores. The stationary state is reached when

(54) $$p_{2}\left(z_{w}+L_{w}\right)=p_{1}\left(z_{w}\right)e^{-\frac{MgL_{w}}{k_{B}T\left(z_{w}\right)}}.$$

This means that the pressures on both sides of the wall satisfy the jump given by the hydrostatic pressure drop inside the column of gas in the wall. When the expressions in the first and second brackets vanish, we find the condition for the position of the cylinder inside the whole column:

(55) $$p_{1}\left(z_{w}\right)=\frac{M_{w}g}{A\left(1-e^{-\frac{MgL_{w}}{k_{B}T\left(z_{w}\right)}}\right)}.$$

The above equation, for the stationary state, binds the mass of the wall, its thickness, temperature, and height above the ground through the pressure at $p_{1}\left(z_{w}\right)$. We see that the pressure needed to squeeze an appropriate amount of gas into the pore depends on the wall’s temperature. A warm wall requires more gas.

Finally, we expand the fraction in (55) assuming that $\frac{MgL_{w}}{k_{B}T\left(z_{w}\right)}\ll 1$ and substitute $p_{1}\left(z_{w}\right)=\rho_{1}\left(z_{w}\right)\frac{k_{B}T\left(z_{w}\right)}{M}$ to find

(56) $$p_{1}\left(z_{w}\right)=\rho_{1}\left(z_{w}\right)\frac{k_{B}T\left(z_{w}\right)}{M}=\frac{M_{w}g}{A\left(1-e^{-\frac{MgL_{w}}{k_{B}T\left(z_{w}\right)}}\right)}=\frac{M_{w}g}{A}\frac{k_{B}T\left(z_{w}\right)}{MgL_{w}}+\ldots$$

and Archimedes’ principle emerges:

(57) $$\rho_{1}\left(z_{w}\right)L_{w}A\;=M_{w}.$$

It states that a body submerged in water will rest exactly where the weight of the displaced fluid is equal to that of the submerged body. Here, equivalently, punching another pore through the wall will not make it move.

## 4. Volumetric Heating of an Ideal Gas Separated by an Adiabatic Wall

**Volumetric heating.** We consider a system similar to the one in Section 2 but with volumetric heating, [16] and start by describing a single compartment. The additional energy supplied uniformly throughout the volume is λ per unit of time and unit of volume. It extends the set of parameters controlling the non-equilibrium steady state of this system to $\left(T_{1},T_{2},\lambda ,V,N\right)$. In the steady state, the pressure *p* and, consequently, the energy density ϵ=(f/2)p are constant, similar to the systems discussed in Section 2. For volumetric heating, the outflow of the heat is balanced by the absorption of heat throughout the system. Therefore, the temperature profile is obtained from the following local energy continuity equation:

(58) $$-\kappa \nabla^{2}T\left(\vec{r}\right)=\lambda ,$$

which assumes Fourier’s law of heat conduction. The coefficient κ is the thermal conductivity, which we assume to be temperature-independent. We apply the mapping of a non-equilibrium steady-state system to a homogeneous equilibrium system. Solving Equation (58) for the temperature profile with the boundary conditions $T\left(z=0\right)=T_{1}$ and $T\left(z=L\right)=T_{2}$, we find

(59) $$T\left(z\right)=-\frac{\lambda }{2\kappa }z^{2}+\left(\frac{T_{2}-T_{1}}{L}+\frac{\lambda L}{2\kappa }\right)z+T_{1}.$$

After the mapping procedure given by Equation (3), we obtain the effective temperature

(60) $$T^{*}=T_{1}F\left(\frac{T_{2}}{T_{1}},\frac{\lambda L^{2}}{\kappa T_{1}}\right),$$

where F(u,w) is a dimensionless function given by

(61) $$F\left(u,w\right)=\frac{\sqrt{{\left(u-1+w/2\right)}^{2}+2w}}{2\left(\tanh^{-1}\frac{u-1+w/2}{\sqrt{{\left(u-1+w/2\right)}^{2}+2w}}-\tanh^{-1}\frac{u-1-w/2}{\sqrt{{\left(u-1+w/2\right)}^{2}+2w}}\right)}.$$

In the limit λ→0, the effective temperature $T^{*}$ reduces to the value given by Equation (5) for an ideal gas in the heat flux induced by the temperature gradient $T_{2}-T_{1}$ (This can be seen by using the definition of inverse hyperbolic tangent in terms of logarithms). On the other hand, if the temperatures on both outer walls are equal (i.e., $T_{2}=T_{1}$), the system reduces to that described in our previous article [16]. The entropy $S^{*}\left(U,V,N\right)$ is given by Equation (14) with $U\left(S^{*},V,N\right)=\left(f/2\right)Nk_{B}T^{*}=\left(f/2\right)pV$. For a fixed number of molecules *N*, the energy change is determined by the control parameters $\left(T_{1},T_{2},\lambda ,V\right)$ through Equation (60). For an incremental and slow change between steady states that does not disturb the pressure uniformity in the system, we have [35]

(62) $$dU=đQ-pdV\;\mathrm{and}\;đQ=T^{*}dS^{*}.$$

**Figure 3:**
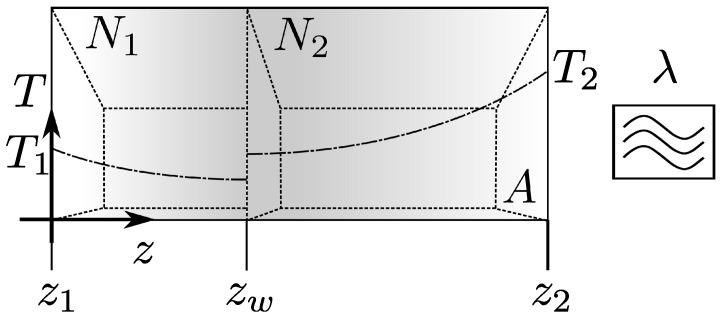
Schematic of a gas confined between two parallel walls of large area A subjected to a homogeneous external energy input of density λ. The external wall located at $z_{1}=0$ is maintained at a temperature $T_{1}$, while the external wall located at $z_{2}=L$ is maintained at a temperature $T_{2}>T_{1}$. The mobile, thin internal wall is adiabatic, resulting in a jump in the temperature profile.

**Movable wall.** We introduce a constraint into the system in the form of a movable adiabatic wall parallel to the bounding walls located at $z=z_{w}$ (see Figure 3). The adiabatic wall does not conduct heat, yet it allows for momentum transport [1]. In the microscopic picture, both are tied to molecular motion, and the mechanism of the position equilibration of the wall depends on the statistics of the thermal collisions on both sides of the wall. In the case of infinite compartments with equal pressure *p* but different temperatures, the wall surprisingly moves toward the hotter region [43]. The molecular collision mechanism is crucial in the case of an array of compartments separated by moving adiabatic walls that fill the space between two fixed walls kept at different temperatures. As a result of its existence, we obtain Fourier’s law [44]. Here, although it moves, we treat the adiabatic wall according to the macroscopic picture. The stable position is set by pressure equality on both sides, and the temperature profile has an additional boundary condition, i.e., ∂T(z)/∂z=0, at the internal wall. Such a wall does not allow for energy transport between compartments as a result of molecular collisions.

The temperature profiles $T^{\left(1\right)}\left(z\right)$ and $T^{\left(2\right)}\left(z\right)$ for subsystems (1) and (2) with $N_{1}$ and $N_{2}$ molecules, respectively, are

(63) $$\begin{array}{cc} \; & T^{\left(1\right)}\left(z\right)=-\frac{\lambda }{2\kappa }{\left(z-z_{w}\right)}^{2}+\frac{\lambda }{2\kappa }z_{w}^{2}+T_{1},\\ \; & T^{\left(2\right)}\left(z\right)=-\frac{\lambda }{2\kappa }{\left(z-z_{w}\right)}^{2}+\frac{\lambda }{2\kappa }{\left(L-z_{w}\right)}^{2}+T_{2}. \end{array}$$

The mapping procedure yields

(64) $$\begin{array}{cc} \; & T_{1}^{*}=T_{1}G\left(\frac{\lambda z_{w}^{2}}{\kappa T_{1}}\right),\\ \; & T_{2}^{*}=T_{2}G\left(\frac{\lambda {\left(L-z_{w}\right)}^{2}}{\kappa T_{2}}\right), \end{array}$$

where

(65) $$G\left(w\right)=\frac{\sqrt{w(w+2)}}{2\tanh^{-1}\sqrt{\frac{w}{w+2}}}.$$

Thus, the energy of the subsystems of $N_{1}$ and $N_{2}$ particles under the constraint is

(66) $$U=U_{1}+U_{2}=\frac{f}{2}N_{1}k_{B}T_{1}G\left(\frac{\lambda z_{w}^{2}}{\kappa T_{1}}\right)+\frac{f}{2}N_{2}k_{B}T_{2}G\left(\frac{\lambda {\left(L-z_{w}\right)}^{2}}{\kappa T_{2}}\right),$$

with $N_{1}+N_{2}=N$.

Our second law of non-equilibrium thermodynamics states that

(67) $$dU_{1}+dU_{2}-\;đQ_{1}-\;đQ_{1}=-\left(p_{1}-p_{2}\right)dV_{1}\leq 0\;,$$

and, therefore, the condition for the whole system to reach a steady state is that the pressure exerted by each subsystem is equal. This condition is equivalent to

(68) $$\frac{1}{z_{w}}G\left(\lambda^{*}{\tilde{z}}_{w}^{2}\right)=r\frac{N_{2}}{N_{1}}\frac{1}{1-{\tilde{z}}_{w}}G\left(\frac{\lambda^{*}}{r}{\left(1-{\tilde{z}}_{w}\right)}^{2}\right),$$

where ${\tilde{z}}_{w}=z_{w}/L$, *r* is the temperature ratio $r=T_{2}/T_{1}$, and $\lambda^{*}=\lambda /\left(\kappa T_{1}\right)$. At equilibrium, i.e., for r=1 and $\lambda^{*}=0$, the internal wall is located precisely in the middle of the system at $z_{w}=0$. For $\lambda^{*}=0$ but r>1, the position of the internal wall is given by Equation (25).

**Figure 4:**
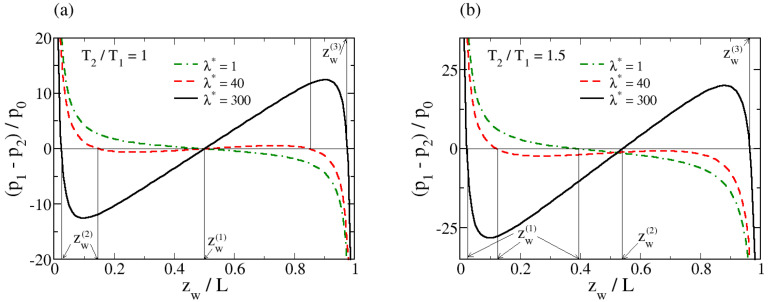
The difference between the pressures in the two subsystems, normalized by the equilibrium pressure $p_{0}=Nk_{b}T_{1}/V$, where $T_{1}$ is the temperature of the external wall located at $z_{1}=0$, as a function of $z_{w}$ for three values of λ. In (**a**), the temperatures at both external walls are equal ($r=T_{2}/T_{1}=1$). In (**b**), the temperatures at both external walls are different ($r=T_{2}/T_{1}=1.5$). The vertical lines mark the positions of the steady states. In (**a**), these are $z_{w}^{\left(1\right)}$ for $\lambda^{*}=1$, and $z_{w}^{\left(1\right)},z_{w}^{\left(2\right)}$, and $z_{w}^{\left(3\right)}$ for $\lambda^{*}=40$ and 300. In (**b**), they are $z_{w}^{\left(1\right)}$ for $\lambda^{*}=1$ and 40, and $z_{w}^{\left(1\right)},z_{w}^{\left(2\right)}$, and $z_{w}^{\left(3\right)}$ for $\lambda^{*}=300$. Here, $\lambda^{*}=\lambda L^{2}/\left(\kappa T_{1}\right)$.

An analysis of Equation (68) shows that the number of solutions for $z_{w}$ varies from one to three depending on the values of $\lambda^{*}$ and *r*. A typical course of the variability of the function $p_{1}\left(z_{w}\right)-p_{2}\left(z_{w}\right)$ is shown in Figure 4, compared to the case of no temperature gradient (r=1). For r=1 and small values of $\lambda^{*}$, the curve $p_{1}\left(z_{w}\right)-p_{2}\left(z_{w}\right)$ is a monotonically decreasing function with a single zero-crossing point at the midpoint of the system. A local stability analysis shows that this is a stable position of the internal wall. Upon increasing the volumetric heating, the function develops a minimum and a maximum but remains symmetrical with respect to $z_{w}=1/2$ with three zero-crossing points at $z_{w}^{\left(1\right)}=0$, $z_{w}^{\left(2\right)}=z_{w}^{*}$, and $z_{w}^{\left(3\right)}=L-z_{w}^{*}$, as illustrated in Figure 4a. Among these three solutions, further analysis shows that locally, both $z_{w}^{\left(2\right)}$ and $z_{w}^{\left(3\right)}$ are stable, whereas $z_{w}^{\left(1\right)}$ is unstable. Moreover, the work required to change the position of the wall from $z_{w}^{*}$ to $L-z_{w}^{*}$

(69) $$W_{z_{w}^{\left(2\right)}->z_{w}^{\left(3\right)}}=-\int_{z_{w}^{\left(2\right)}}^{z_{w}^{\left(3\right)}}\left(p_{1}\left(z\right)-p_{2}\left(z\right)\right)dz$$

is equal to zero ($W_{z_{w}^{\left(2\right)}->z_{w}^{\left(3\right)}}=0$). This, in turn, means that these two stable steady states coexist.

In the case of a non-zero temperature gradient, symmetry is broken, and the situation changes qualitatively. The single solution that exists for smaller values of $\lambda^{*}$ shifts away from the midpoint toward the lower temperature wall, as might be expected. The difference $p_{1}\left(z\right)-p_{2}\left(z\right)$ becomes non-monotonic as $\lambda^{*}$ increases, similar to the behavior observed in the absence of a temperature gradient. Initially, the extremes of the function develop below the y=0 axis. Above the critical value $\lambda_{c}^{*}\left(r\right)$, at which the maximum of the function $p_{1}\left(z\right)-p_{2}\left(z\right)$ touches the y=0 axis, three zero crossing points $z_{w}^{\left(1\right)}<z_{w}^{\left(2\right)}<z_{w}^{\left(3\right)}$ appear, as shown in Figure 4b. These correspond to three non-equilibrium steady states. To evaluate their stability, we assume that the internal wall is displaced from a specific steady state in both directions. The pressure difference will push the inner wall toward $z_{w}^{\left(1\right)}$ or $z_{w}^{\left(3\right)}$ but push it away from $z_{w}^{\left(2\right)}$. Thus, the middle position is locally unstable, whereas both positions close to the external walls are locally stable. Considering the work needed to move the wall from position $z_{w}^{\left(1\right)}$ to position $z_{w}^{\left(3\right)}$ allows us to determine which steady state is globally stable. If the work $W_{z_{w}^{\left(1\right)}->z_{w}^{\left(3\right)}}$ done on the system during this process is negative, the final position $z_{w}^{\left(3\right)}$ corresponds to a globally stable steady state. If this work is positive, the initial position $z_{w}^{\left(1\right)}$ is globally stable. We calculate this work numerically up to machine accuracy. We find that for r>1, due to the asymmetry, the work is positive for all values of λ. Therefore, $z_{w}^{\left(1\right)}$ is always the globally stable steady-state position of the internal wall. Thus, the internal wall is globally stable when moved closer to the colder external wall.

## 5. Couette Flow of an Ideal Gas

**Figure 5:**
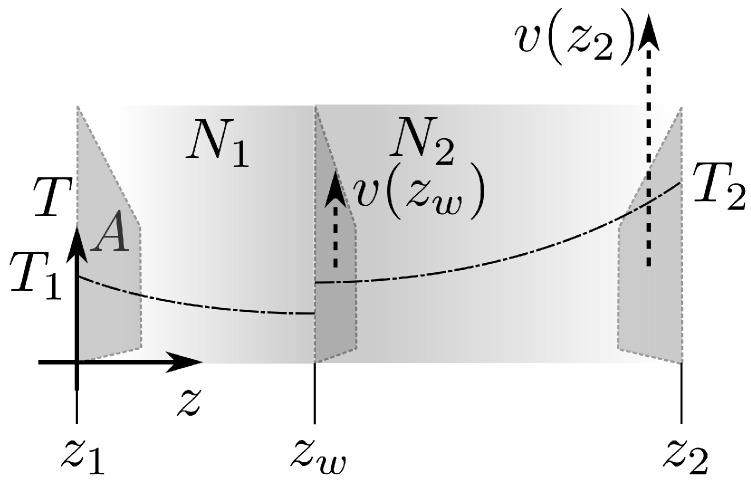
Schematic of a gas confined between two parallel moving walls of large area A. The external wall located at $z_{1}=0$ is fixed and is kept at a temperature $T_{1}$, while the external wall located at $z_{2}=L$ is moving with velocity $v_{2}$ and kept at a temperature $T_{2}>T_{1}$. The mobile, thin internal wall is adiabatic, resulting in the temperature profile jump.

The last example is the Couette flow of an ideal gas [38]. The gas flows between two walls, as shown in Figure 5. The wall at $z_{2}=L$ moves at a constant speed $v_{2}$ in the direction of the imposed shear force. The wall at $z_{1}=0$ is fixed. The velocity profile is linear: $v\left(z\right)=v_{2}z/L$. Inside the system there is an adiabatic massless wall at location $z_{w}$, which moves at the local speed of the fluid $v_{w}=v_{2}z_{w}/L$ in the same direction as the upper wall. The wall divides the system into two parts: 1 and 2. The location of the wall is an internal constraint in the system. We ask the following question: if we allow the wall to move along the *z* direction, what will be the final stationary location of this internal wall? This system differs from the previous ones in two ways. Firstly, the total energy includes both internal energy and kinetic energy. The latter was absent in the cases discussed in the previous paragraphs. Secondly, the system exchanges energy with the external world in the form of heat and work. Continuous input of work maintains a constant flow and constant kinetic energy despite dissipation due to shear. In the steady state, with the internal wall fixed in place, the work dissipates as heat within the system. When we release the internal wall, it starts moving in the *z* direction. During its motion, the external machine must perform extra work to overcome extra shear (the constant velocity of the upper wall) and dissipation.

The internal wall divides the system into two subsystems: 1 and 2. According to the equations of irreversible thermodynamics [38], the change in internal energy $dU_{i}$ for subsystem i(i=1,2) is given by

(70) $$dU_{i}=đQ_{i}+đW_{i}+đD_{i},$$

where $đQ_{i}$ is the excess heat in subsystem *i* (i=1,2), $đD_{i}$ is the excess dissipation in subsystem *i*, and $đW_{i}=-p_{i}dV_{i}$ is the volumetric work performed during the transition in subsystem *i*. In this non-equilibrium case, we have $T^{*}dS^{*}=đQ+đD$. Thus, the entropy change is due to both excess heat exchange and dissipation due to shear within the system. $dV_{i}$ is the change in volume in subsystem *i*. From the equation of irreversible thermodynamics [38], it follows that the change in kinetic energy, $E_{k,i}$ in subsystem *i* (i=1,2) has the following form:

(71) $$dE_{k,i}=đW_{w,i}-đD_{i}.$$

The excess work performed by the surface forces, $đW_{w,i}$, during the motion of the internal wall in the *z* direction maintains a constant velocity profile in the system despite changes in the shear forces at the walls. Again, motivated by the second law of equilibrium thermodynamics, we generalize the second law as follows: From the sum of the total energy of the two subsystems, we subtract the heat and work exchanged with the environment during the processes and require that the difference be non-positive:

(72) $$\begin{array}{cc} dU_{1}+dU_{2}+dE_{k,1} & +dE_{k,2}-đQ_{1}-đQ_{2}\\ \; & -đW_{w,1}-đW_{w,2}\leq 0. \end{array}$$

From this equation, we obtain the expected form of the second law:

(73) $$-(p_{1}-p_{2})dV\leq 0.$$

The equality in the above expresion defines the condition for the stationary position of the wall, given by the equality of pressures $p_{1}=p_{2}$ in the two subsystems.

## 6. Discussion

The system at equilibrium, which exchanges heat with the environment, satisfies the inequality dU−đQ≤0 at a constant temperature. In this contribution, we showed that the same inequality holds for non-equilibrium states with constant boundary conditions, including boundary temperature. This inequality is the second law of non-equilibrium thermodynamics for systems coupled to the environment via heat flux.

In equilibrium thermodynamics, at constant temperature *T* and pressure *p*, the second law states that dU−TdS−pdV≤0. We can write it in a more general form as dU−đQ−đW≤0. In the example of the Couette flow, a similar inequality sets the direction of spontaneous processes in the system. In general, in a system that exchanges energy via heat đQ and different forms of work $đW_{j}$, the following inequality should govern spontaneous processes:

(74) $$dE-đQ-\sum_{j}đW_{j}\leq 0.$$

*E* is the system’s total energy, including internal, potential, and kinetic energy. The most challenging part of our study was identifying the various terms in this equation and calculating the net heat and work performed during the process.

We are now in a position to discuss the second law in more detail, including all its subtleties. The second law of equilibrium thermodynamics states that the entropy of an isolated system reaches its maximum value at equilibrium. However, S(U,V,N) has a well-defined value for fixed U,V,N. Therefore, we need to compare the entropy of the system in its equilibrium state to the entropy of the system in states that are not reached simply by changing the state parameters U,V,N. We introduce an extra parameter *x*, usually in the form of a constraint in the system (like a movable wall), and state that S(U,V,N,x) is maximized as a function of *x* for fixed state parameters U,V,N (internal energy, volume, and number of particles, respectively). Similarly, when the system has a constant temperature, the Helmholtz free energy F(T,V,N,x) is minimized as a function of *x* (the variable describing the internal constraint) at constant T,V,N (temperature, volume, and number of particles, respectively). We treat this extra parameter as arising from an external device, i.e., an external device that performs work on the system by coupling to this internal parameter *x* and moving the system in a reversible manner (i.e., via a sequence of equilibrium states) between states that are not accessible by simply changing T,V,N. Let us define the work performed on the system by this external device as $đW_{z}$. In the process of transitioning between states, the first law of thermodynamics must be obeyed (conservation of energy). Thus, we have $dU=đQ+đW_{z}$. the second law states that if we move a system from a stable state to a less stable state, the external device will perform work on our system, i.e., $đW_{z}>0$. On the other hand, if we move from a less stable state to a more stable state, the system will perform work on the device, and $đW_{z}\leq 0$. This inequality is yet another statement of the second law. For example, in the case of the movable wall, we can apply an external force to the wall and move the system between states, where the pressures on both sides of the internal wall are different and satisfy the equality $p_{1}-p_{2}=F/A$, where A is the area of the wall and *F* is the external force. Now, we can rewrite the first law of thermodynamics in the following form: $dU-đQ=đW_{z}\leq 0$, and thus obtain dU−đQ≤0. This form is more convenient than $đW_{z}\leq 0$ because we do not need to create a new device for each case or introduce new parameters. Everything can be calculated from the system’s state if we know dU and đQ. However, the form dU−đQ compares neighboring states and is therefore local. In non-equilibrium states, this form is insufficient for predicting the direction of spontaneous processes. In many non-equilibrium situations, there may be many stable local states. For example, in Section IV, we described the volumetric heating of a gas. There, we identified three different states that satisfied dU−đQ=0. The question is how to compare these states and determine which is the most stable. We propose in this paper to calculate the total work performed along the path, i.e., $\int đW_{z}$. If the total work is negative, it means that we have moved from a less stable state to a more stable state. Thus, the second law would be ∫(dU−đQ)≤0. Because work depends on the chosen path, we additionally require that this work be maximal, i.e., max∫(dU−dQ)≤0. The calculations must be performed under constant boundary conditions. In simple terms, the second law states that the direction of a spontaneous process involves removing some energy from the system by performing work on an external device that keeps the system in a less stable state.

This contribution, together with our previous works on the first law of non-equilibrium thermodynamics, constitutes a good starting point for applying the presented second law of non-equilibrium thermodynamics to systems undergoing chemical reactions in photo-reactors and flow reactors, Rayleigh-Benard convection, thermoosmosis, and, finally, lift force in hydrodynamic flows.

## Data Availability

Data is contained within the article.
